# The diabetes insulin self-management education (DIME) intervention for people with type 2 diabetes starting insulin: a pilot feasibility randomised controlled trial

**DOI:** 10.1186/s40814-023-01318-x

**Published:** 2023-05-26

**Authors:** Kirsty Winkley, Taru Sorsa, Qingxiu Tian, Ilse Reece, Christina Fitzgerald, Mark Chamley, Khalida Ismail, Angus Forbes, Rebecca Upsher

**Affiliations:** 1 Florence Nightingale Faculty of Nursing, Midwifery & Palliative Care, King’s College London, SE1 8WA London, UK; 2 Institute of Psychiatry, Psychology & Neuroscience, King’s College London, London, UK; 3 Department of Endocrinology, The First Affiliated Hospital of Shandong First Medical University & Shandong Provincial Qianfoshan Hospital, Jinan, China; 4 Lambeth Diabetes Intermediate Care Team, London, UK

**Keywords:** Type 2 diabetes, Insulin, Diabetes self-management, Pilot study, Intervention

## Abstract

**Objective:**

To determine the feasibility and acceptability of a diabetes insulin self-management education (DIME) group intervention for people with type 2 diabetes starting insulin.

**Design:**

Single-centre parallel randomised pilot trial.

**Setting:**

Primary care, South London, UK.

**Subjects:**

Adults with type 2 diabetes, requiring insulin treatment, on maximum tolerated dose of 2 or more oral antidiabetic drugs with *HbA1c* > / = 7.5% (58 mmol/mol) on 2 occasions. We excluded people who were non-fluent in English; morbid obesity (BMI > / = 35 kg/m^2^); in employment that contraindicates insulin treatment; and those with severe depression, anxiety disorders, psychotic disorders, personality disorders, or cognitive impairment.

**Methods:**

Participants were randomised using blocks of 2 or 4 to 3, 2-h group, face-to-face, DIME sessions or standard insulin group education sessions (control). We assessed feasibility according to consent to randomisation and attendance at intervention (DIME) and standard group insulin education sessions. Acceptability of the interventions was determined using exit interviews. We additionally measured change in self-reported insulin beliefs, diabetes distress and depressive symptoms between baseline and 6-month post-randomisation.

**Results:**

There were 28 potentially eligible participants, of which 17 consented to randomisation, 9 were allocated to the DIME group intervention and 8 were allocated to the standard group insulin education. Three people withdrew from the study (1 from DIME and 2 from standard insulin education) before the start of the first session and did not complete baseline questionnaires. Of the remaining participants (*n* = 14), all DIME participants (*n* = 8) completed all 3 sessions, and all standard insulin education participants (*n* = 6) completed at least 1 standard insulin education session. The median group size was 2, the mean age of participants was 57.57 (*SD* 6.45) years, and 64% were female (*n* = 9). Exit interviews demonstrated that all participants (*n* = 7) found the group sessions acceptable, and thematic analysis of interview transcripts indicated social support, the content of group sessions and post-group experiences were positive, especially amongst DIME participants. There was improvement on self-report questionnaires.

**Conclusions:**

The DIME intervention was acceptable and feasible to deliver to participants with type 2 diabetes starting insulin in South London, UK.

**Trial registration:**

International Study Registration Clinical Trial Network (ISRCTN registration number 13339678).

## Key messages regarding feasibility

- What uncertainties existed regarding feasibility?
  - ◦ It was not known whether the group DIME intervention was feasible and acceptable for adults with type 2 diabetes starting insulin.
- What are the key feasibility findings?
  - ◦ It was not possible to recruit the planned sample size due to COVID-19. However, intervention participants found DIME to be acceptable, and it was feasible to conduct a randomised controlled trial.
- What are the implications of the feasibility findings for the design of the main study?
  - ◦ As the DIME intervention was found to be acceptable and feasible this would warrant a full-scale trial to determine its effectiveness. However, further development leading up to a trial and ongoing process evaluation is important to determine the longer-term implications caused by COVID-19.

## Introduction

### Scientific background and rationale

Many people require insulin therapy within 5–10 years of type 2 diabetes (T2D) diagnosis [1, 2]. For those on maximum doses of 2–3 oral antidiabetic drugs (OAD), insulin remains the next step to optimising glycaemia [1, 2]. Compared with insulin, newer incretin analogues are expensive, are unsuitable for people with some comorbidities, and there is less long-term safety data [1]. In the United Kingdom (UK), the average duration of diabetes is 8.5 years with hyperglycaemia (HbA1c 84 mmol/mol) [3] before insulin is first prescribed, suggesting a significant, up to 5 years delay, which brings forward the risk of diabetes complications. The literature recognises organisational, health provider and patient factors contributing to this inertia in insulin treatment [4–6].

In the UK, there are ~ 4.7 million people with diabetes, 90% have T2D, and approximately 30–40% with T2D are either prescribed or require insulin [3, 7]. In a study of 219,000 adults with T2D on the UK Primary Care Clinical Practice Research Datalink database in 2019, 15% (95% *CI* 14.1–15.9) were prescribed insulin as monotherapy, and for dual and triple therapy, 25.5% (95% *CI* 25.5–26.4) and 16.2% (95% *CI* 15.7–16.7), respectively [8]. It is estimated that at least 1/3rd do not take insulin as prescribed [9], which wastes National Health Service (NHS) funds. The cost of insulin to the NHS is estimated at £360 million annually [10].

Many people with T2D do not want insulin because they worry about weight gain, hypoglycaemia, and painful injections and perceive their diabetes has progressed [5]. Qualitative studies with people from non-Western countries/ethnic minorities describe misconceptions regarding insulin and diabetes, injection fear [11, 12], distrust of Western medicine [13], inconvenience, little family support [14], high-cost, religious beliefs [13], social stigma and body image [15] as barriers. This suggests that ethnicity and cultural factors are relevant in insulin self-management. Cross-sectional studies of insulin-treated T2D versus insulin-naïve people have found differences in psychological and social factors, such as people who are obese or overweight or have jobs where insulin is contraindicated are more negative about insulin [16, 17]. Likewise, psychological measures of insulin-specific beliefs, often labelled ‘psychological insulin resistance’, are more common in insulin naïve cohorts [18]. Some studies report that depressive symptoms and diabetes-specific distress are associated with negative appraisals of insulin [19]. However, these beliefs are modifiable, and 2 studies suggest that negative insulin beliefs may improve after insulin initiation [20, 21].

Skills for supporting people with starting insulin used to be the preserve of hospital diabetes clinics, but the growing population of T2D means this no longer provides value. National policy has governed the shift of diabetes provision into primary care. Some evidence suggests insulin start groups are effective and cost-effective [22]. In a systematic review and meta-analysis, we conducted of 10 studies of insulin education delivered by diabetes specialists in primary care/outpatient settings; there was a statistically significant improvement in glycaemic control for intervention participants. However, most studies were of poor quality [23]. Despite this, no national or international curricula include skills/techniques/competencies required to address the health beliefs, fears and worries in accepting insulin as part of diabetes self-management [24].

The diabetes insulin self-management education (DIME) intervention study aims to reduce the negative psychological impact of insulin treatment and improve confidence in insulin self-management. Optimising the effectiveness of group interventions may reduce the cost to the NHS on wasted prescriptions and improved health and take advantage of informal social support around us [25]. Therapies such as motivational interviewing (MI) successfully addressing concerns by prompting dialogue to reduce ambivalence to behaviour change, and self-management in people with T2D [26], were the underpinning psychological skill to deliver the DIME intervention. The main aims of this study were to (1) determine the feasibility and acceptability of delivering the group DIME intervention for people with T2D starting insulin in South London, UK, and (2) to determine outcome data on glycaemic control and psychological outcomes, including insulin beliefs (measured using the Insulin Treatment appraisal Scale, ITAS), which may inform a sample size calculation for a future randomised controlled trial.

## Methods

### Design

This study was a single-centre parallel randomised pilot trial with 1:1 allocation conducted in South London, UK. The study protocol was registered with the International Study Registration Clinical Trial Network (ISRCTN registration number 35501). NHS Health Research Authority ethical approval was obtained (17/LO/0363).

### Participants

Inclusion criteria were diagnosis of T2D, aged 40 + years, identified by diabetes team or general practitioner (GP) as requiring insulin treatment, i.e. on maximum tolerated dose of 2 OADs and glycaemic control HbA1c > / = 7.5% (58 mmol/mol) on 2 occasions.

Exclusion criteria were nonfluency in English; morbid obesity (*BMI* > / = 35 kg/m^2^); in employment that contraindicates insulin treatment, e.g. heavy goods vehicle (HGV) driver; and those with severe depression, anxiety disorders, psychotic disorders, personality disorders or cognitive impairment.

### Settings

The first setting was in the South London Borough of Lambeth. The Lambeth Diabetes Intermediate Care Team (LDICT) is an intermediate diabetes service supporting GPs and practice nurses to optimise the treatment of people with T2D. GPs and practice nurses refer patients to the service when they require insulin or injectable therapy. LDICT has an existing group insulin education programme. There are 4 clinic sites across the borough, and at the time of the study starting, 2 of which hosted the insulin education programme.

The second setting for recruitment was the community diabetes team for the South London Borough of Lewisham. This was planned to start in June 2020 but did not go ahead because study recruitment was stopped early because of COVID-19. Lewisham has a community diabetes service to which GPs and practice nurses can refer patients for optimisation of T2D. Lewisham diabetes offers patient appointments within the main diabetes outpatient service at University Hospital Lewisham and a community site. Lewisham does not run group insulin starts, and diabetes nurses conduct insulin starts on a one-to-one basis.

### Recruitment

Participants were identified from LDICT by the diabetes clinical team. All potentially eligible participants who had been referred to the group insulin education programme were invited by an LDICT clinician to participate in the study and given a study pack including a participant information sheet, consent form and baseline questionnaires. Once the participant consented, the researcher (an LDICT diabetes nurse [KW]) telephoned them and gave more study information with the opportunity to ask questions. When participants arrived for the first insulin start group session, they were randomised to either DIME or the usual insulin start group (ISG).

### Interventions

For all participants, insulin was prescribed by a doctor or nurse prescriber.

Participants who were randomised to the control group received standard group insulin start with a diabetes nurse (IR or CF), 1–2 h at week 1 and week 3. Control group participants did not receive a face-to-face 3rd session but were followed up by telephone at least once within 3 months of randomisation following the current treatment model in Lambeth. Session 1 covered the following topics: patient stories, self-injection of the first dose, safe insulin administration, storage, dose titration, hypoglycaemia, driving, employment and blood glucose monitoring. Session 2 covered insulin dose titration, sick day rules, holidays, diet and alcohol. Standard leaflets were given to participants: TREND hypoglycaemia, driving and travel; the manufacturer’s leaflet for the insulin pen device prescribed, e.g. Kwikpen; and an NHS insulin passport with details of prescribed insulin and start date. Participants had access to telephone follow-up as needed. An overview of the control group sessions can be seen in Table 1.

**Table 1:** Standard insulin start group content (control) and DIME

Session	Current insulin start group content	DIME content
**Session 1**	• Introductions	• Introductions
	• Ground rules	• Ground rules
	• Initial questions	• Exploring perspectives
	• Diabetes & the need for insulin	• Injection technique and checking blood glucose
	• Safe insulin administration	• Activity 1: Decisional balancing tool
	• Insulin storage	• Diabetes medication choice
	• Self-injecting first dose	• What is diabetes and the need for insulin?
	• Insulin dose titration	• Activity 2: What’s in it for you?
	• Activity 1: Dose titration	• Hypoglycaemia
	• Diabetes medications	• Dose titration
	• Hypoglycaemia	• Activity 3: Dose titration
	• Driving with insulin and employment	• Driving and insulin
	• Blood glucose meter technique review	• Techniques for remembering diabetes medication
		• Activity 4: Goal setting
**Session 2**	• Reflections & titration issues	• Introductions
	• Activity 1: Quiz of last session	• Review of progress since last time
	• Problems encountered	• Activity 1: Wheel of change
	• Sick day rules	• Relapses
	• Travel	• Feedback on blood glucose reading and goal setting
	• Annual review & interpreting results	• Activity 2: Quiz of last session
	• Next steps	• Complications
	• Burning issues	• HbA1c targets
		• Activity 3: Changes in blood glucose
		• Activity 4: Exploring importance of insulin
		• Sick day rules
		• Diabetes technology
		• Activity 5: Maintenance plan
**Session 3**	N/A	• Introductions
		• Review of progress since last time
		• Feedback on blood glucose readings and goal setting
		• What are carbohydrates?
		• Activity 1: Carb counting knowledge quiz
		• Weight, insulin & diabetes
		• Exercise and diabetes
		• How many carbs?
		• Types of carbs and glycaemic index
		• Ways to improve blood glucose
		• Activity 2: Thinking about carbs in our diet
		• Time restricted eating
		• Benefits of weight loss
		• Insulin on holiday

Participants randomised to DIME received face-to-face group sessions for 1–2 h on week 1 and week 3. They received a 3rd session around 6 weeks later (within 3 months of randomisation). All sessions were manualised and facilitated by the researcher (KW), a diabetes nurse trained in motivational interviewing. Sessions were audio-taped when participants gave consent. Sessions 1 and 2 included standard Lambeth insulin programme topics as listed above. Still, the sessions were adapted using psychological techniques to address worries and concerns around insulin and gave them the knowledge and skills to inject and self-manage insulin safely. The aim was to increase the perceived value of making changes by eliciting patients’ views and reinforcing change talk. Sessions also aimed to support behaviour change within a social context. The 3rd session focussed on diet and exercise, topics not currently covered in detail in the standard insulin start programme, again delivered according to the underlying psychological model and principles, i.e. social cognitive theory [25] and the capability opportunity motivation-behaviour (COM-B) model [27]. Each session included action planning and goal setting and homework. Printed materials were given to participants to be completed during the sessions as part of group exercises, and educational literature covering topics discussed in each session was given to them to take home. All participants received an NHS insulin passport as above. An overview of DIME can be seen in Table 1. Participants randomised to the DIME intervention in March 2020, the last participants (*n* = 3) before the lockdown in England and the study closed to recruitment received 1 group session. The 2 remaining sessions were delivered individually by telephone with session materials posted.

All participants received a £10 high street voucher for attending the insulin group sessions. All were due to receive a clinical follow-up 3 months following randomisation, and study outcome measurements/questionnaires were issued 6 months post-randomisation. Participants were sent a £10 high street voucher to encourage them to return questionnaires.

### Nurse training

One diabetes nurse (KW) was trained to deliver the DIME intervention using the DIME manual. The intervention was delivered using motivational interviewing principles [28]. Before commencement, the nurse had received advanced training in motivational interviewing and was rated as competent by an independent rater using the motivational interviewing treatment integrity (MITI) tool [28].

The standard insulin start group intervention was delivered by 2 other Lambeth diabetes nurses (IR and CF) who had not received training in motivational interviewing.

### Clinical and questionnaire outcome measures

Sociodemographic, biomedical and psychological measures were taken pre-randomisation.

#### Planned baseline and outcome measures

##### Sociodemographics

Age (years), self-reported sex and ethnicity, educational level (primary, secondary and college/university) and employment status.

#### Outcome measures

##### Biomedical

Weight (kg), BMI (kg/m^2^) and HbA1c (mmol/mol) within 3 months of referral for insulin treatment. Current diabetes treatments (oral antidiabetic agents and injectable therapies), duration of diabetes (years), and complication status were extracted from GP medical records. Complication status included retinopathy (according to the last retinal photograph), nephropathy (positive albumin creatinine ratio or chronic kidney disease), diabetic neuropathy and macrovascular disease (cerebrovascular accident, previous myocardial infarction or revascularisation).

##### Psychological

The main psychological outcome measure was attitudes towards insulin treatment, measured using the insulin treatment appraisal scale (ITAS) [29]. This is a 20-item self-report questionnaire consisting of 16 negatively worded statements and 4 positively worded statements. It is scored from 1 to 5 (strongly disagree to strongly agree). Negative appraisal items are summed to provide a negative insulin beliefs scale with higher scores depicting more negative appraisal of insulin. Positive items are summed to provide a positive appraisal of insulin. Total scale scores with reverse scoring of positive items were calculated. The ITAS is a valid and reliable measure of assessing insulin beliefs in people with T2D. We reported positive and negative scale scores as the latter is more useful at discriminating between insulin users [30]. Other outcome measures included the following: the problem areas in diabetes (PAID) scale, a validated measure used to determine the level of diabetes distress. This is a 20-item self-report questionnaire with items measured on a 5-point scale (0–4, where 0 = not a problem to 4 = serious problem). A score out of 100 is achieved by multiplying the total score by 1.25 [31]. Scores of 40 or more indicate the presence of diabetes distress. The presence of depressive symptoms was measured using the Patient Health Questionnaire 9 (PHQ-9). This 9-item scale aligns with the *Diagnostic and Statistical Manual of Mental Disorders* (DSM) criteria for determining the presence of a depressive episode. Self-reported responses to symptoms of depression in the last 2 weeks are measured on a 4-item scale (0–3) ranging from ‘not at all’ = 0 to ‘nearly every day’ = 3 [32]. Scale scores are summed, and a cut-off of 10 or more is thought to indicate the presence of depressive symptoms.

#### Planned outcome measures at 6 months

The main outcome measure was a change in ITAS score. Secondary outcomes included changes in PAID scores and PHQ9 scores. It was planned to measure a change in weight (kg) and HbA1c (mmol/mol). However, because of UK lockdown restrictions from the end of March 2020, it was impossible to arrange a face-to-face follow-up research appointment to collect the data.

### Pilot trial objectives

The main objectives of the pilot study were to determine the feasibility and acceptability of the DIME intervention for people with T2D starting insulin.

Feasibility was determined by the following:

1. Recruitment rate: To ensure that a fully powered study would be feasible, one of the main aims was to recruit 60 participants to be randomised.
2. Response rate: Based on pre-specified criteria, an uptake > / = 40% would be acceptable.
3. Retention rate: A retention rate of > / = 70% was used.
4. Intervention uptake: Attendance at > / = 50% of sessions

Acceptability of the intervention for participants was assessed using exit interviews as part of the process evaluation.

#### Process evaluation

To evaluate the patients’ experience, exit interviews with participants were conducted and qualitatively analysed. Adherence to the DIME curriculum was measured by a research assistant who observed the DIME sessions. Treatment fidelity in terms of adherence to the principles of motivational interviewing by the nurse delivering the DIME intervention was measured using the motivational interviewing treatment integrity (MITI) tool by an external rater.

### Sample size

The study aimed to recruit 60 patients (30 per treatment condition). It was estimated that this would be achieved by approaching 150 eligible patients and a participation rate of 40% to within a 95% confidence interval of + / − 10%.

### Randomisation

Block randomisation was conducted using block sizes of 2 or 4 by the King’s Clinical Trials Unit (CTU) at King’s Health Partners. Sequence generation was computer generated using a web-based system. The randomisation ratio was 1:1. Recruitment of participants was conducted independently of CTU randomisation to ensure adequate allocation concealment.

### Blinding

It was not possible to blind the interventionists or participants to group allocation. However, baseline self-report questionnaires were administered by a research assistant and completed by participants before group allocation. At 6-month follow-up, questionnaires were mailed to participants. It was not possible to arrange the planned face-to-face study follow-up with a research assistant blind to allocation because of restrictions on research due to COVID-19.

### Analytical methods

Numbers and percentage of potentially eligible participants consented and randomised, attended study sessions and provided follow-up data were recorded. Baseline characteristics of the sample were reported using descriptive statistics, means and standard deviations, numbers and percentages. The mean difference in outcome data between those in the two treatment conditions and 6-month follow-up and the effect size of the difference between means according to group were examined using analysis of covariance (ANCOVA) adjusting for baseline values. An assessment of the assumptions of ANCOVA was made including assessment of the normality of the distribution of residuals, homogeneity of variances using Levene’s test, homoscedasticity using scatterplots of standardised residuals and predicted values and the homogeneity of regression slopes to test for interaction effects. Quantitative data was analysed using IBM SPSS version 28. A qualitative evaluation of participant exit interviews was conducted. Semi-structured interviews were audio-recorded and transcribed verbatim. Data was managed using NVivo 12. Data was analysed according to the thematic framework method [33] by 2 independent researchers (RU and TS).

## Results

### Community site recruitment

The aim was to recruit participants from 2 community diabetes services in South London. Although recruitment of participants was only possible from 1 site, i.e. Lambeth, a second site, Lewisham, had agreed and was ready to start in June 2020. However, participant recruitment for all non-COVID-19 research was paused in the UK from the end of March 2020, so the Lewisham community diabetes team could not participate.

### Feasibility — participant recruitment, response rate, retention and uptake of the intervention

**Fig. 1:**
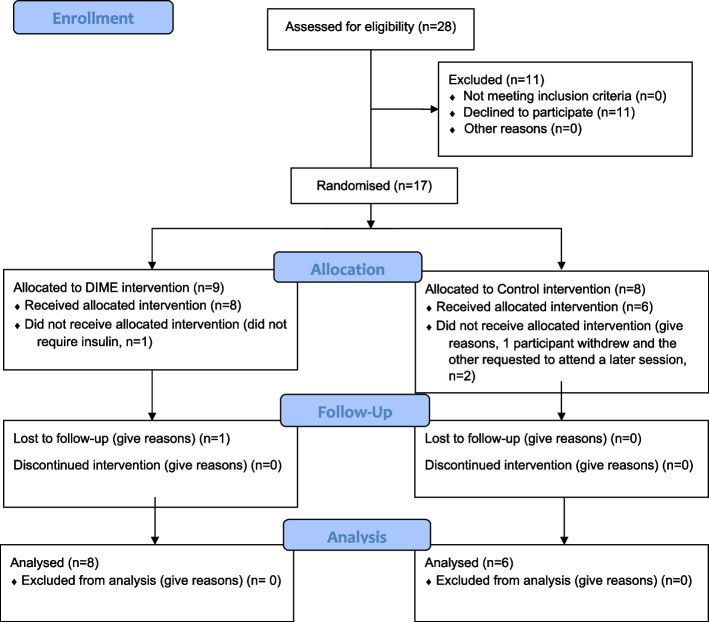
CONSORT 2010 flow diagram for DIME study

Of the 28 people identified as potentially eligible for the study, 17 consented to the study and randomisation, giving a participation rate of 61%. This was more than the 40% minimum participation rate set for the study. However, 3 people withdrew from the study (1 from DIME and 2 from standard insulin education) prior to the start of the first session and did not complete baseline questionnaires. Reasons for withdrawal included the following: no longer needing insulin (*n* = 1), wanting an alternative date and time for appointment (*n* = 1), and declining to give a reason (*n* = 1). Only 17 out of the 60 participants (28%) required for the study were recruited; again, this was owing to COVID-19. For further details, please see CONSORT flow diagram in Fig. 1.

Regarding retention, 13/17 (76%) questionnaires were completed at baseline and 6-month follow-up, with the same proportion attending session 1 of the intervention. All DIME participants, apart from 1 withdrawal, completed all sessions. For the control, all participants except for 2 (the 2 withdrawals) completed all sessions. Therefore, retention and attendance were > 70%.

In terms of feasibility, the recruitment rate was less than planned owing to COVID-19, but response rate, retention and uptake of sessions indicate the feasibility of the study.

### Baseline characteristics and follow-up

The main characteristics of the sample at baseline stratified by group can be found in Table 2. In summary, the mean age of the sample was 58.35 (*SD* 7.11), and 58.8% of the sample was female. The sample was ethnically diverse, 5 people (29.4%) were Caucasian, and the rest (70.6%) were from ethnic minority communities. Ten received secondary/high school education (58.8%), and 9 (52.9%) were in full-time employment.

**Table 2:** Baseline characteristics (including withdrawals)

	**Control (*n* = 8)**	**DIME baseline (*n* = 9)**	**Total (*N* = 17)**
**Mean age, years (SD)**	57.00 (8.25)	59.56 (6.17)	58.35 (7.11)
**Female (%)**	3 (37.5)	7 (77.8)	10 (58.8)
**Ethnicity (%)**			
• White	3 (37.5)	1 (11.1)	4 (23.5)
• Other white	0	1 (11.1)	1 (5.9)
• Pakistani	0	1 (11.1)	1 (5.9)
• Other Asian	2 (25.0)	0	2 (11.8)
• African	0	4 (44.4)	4 (23.5)
• Caribbean	3 (37.5)	2 (25)	5 (22.2)
**Education (%)**			
• Secondary	4 (50)	6 (66.7)	10 (58.8)
• University	4 (50)	3 (33.3)	7 (41.2)
**Employment (%)**			
• Full-time	5 (50)	5 (55.6)	9 (52.9)
• Part-time	1 (12.5)	1(11.1)	2 (11.8)
• Unemployed	1 (12.5)	0	1 (5.9)
• Medically retired	1 (12.5)	0	1 (5.9)
• Retired	1 (12.5)	2 (22.2)	3 (17.6)
• Carer	0	1 (11.1)	1 (5.9)
**Attended structured education (%)**	2 (25.0)	5 (55.6)	7 (50)
**Mean duration of diabetes (SD)**	12.00 (6.00)	12.67 (4.06)	12.35 (4.91)
**OADs (median, range)**	2.0 (1–3)	2.0 (1–3)	2.0 (1–3)
**GLP1-RA (%)**	0	3 (33.3)	3 (17.6)
**Mean HbA1c, mmol/mol (SD)**	99.88 (16.15)	97.56 (17.19)	98.65 (16.22)
**Mean weight, kg (SD)**	83.54 (23.63)	86.32 (19.44)	85.01 (20.86)
**Microvascular disease (%)**			
• Retinopathy	1 (12.5)	5 (55.6)	6 (35.3)
• Nephropathy	1 (12.5)	4 (44.4)	5 (29.4)
• Neuropathy	0	0	0
**Macrovascular disease (%)**	0	2 (22.2)	2 (11.8)

Regarding diabetes variables, 50% of participants had attended structured T2D education before participating in the study. The mean duration of T2D was 12.35 (4.91) years, participants were taking a median of 2 OADs, and 3 people in the DIME group were taking GLP1-RA. The mean HbA1c within 3 months of randomisation was 98.65 mmol/mol (SD 16.22), and the mean weight was 85.01 kg (SD 20.86). Six participants (35.5%) had retinopathy present, 5 (29.4%) had existing nephropathy, none had neuropathy, and 2 had some macrovascular disease (11.8%).

### Potential efficacy of intervention — secondary outcomes

**Fig. 2:**
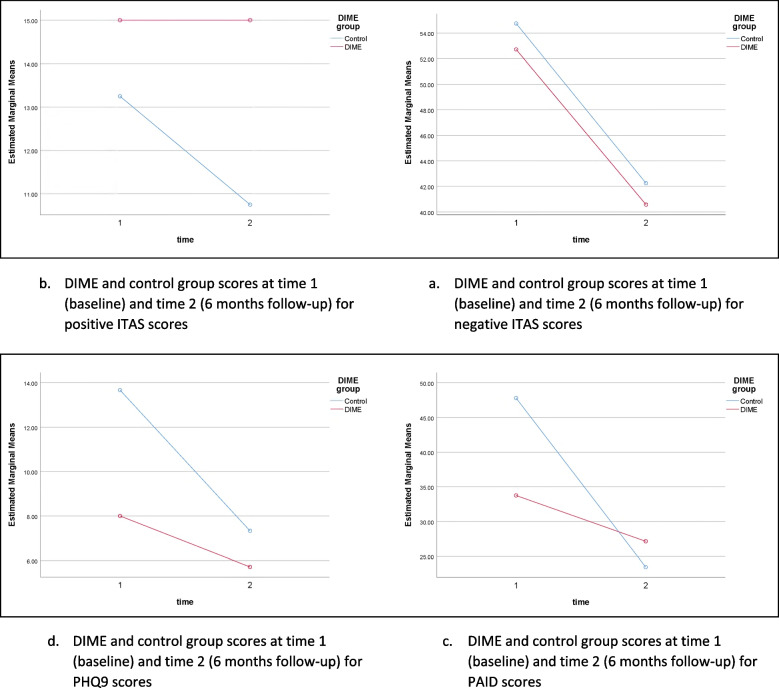
Questionnaire scores at baseline (time 1) and 6-month follow-up (time 2)

Mean change in self-report questionnaires and effect sizes were calculated at 6 months from randomisation. All scores were improved at 6 months regardless of group. Please see Table 3 and Fig. 2.

**Table 3:** Means (SD) of study questionnaires at baseline and 6-month follow-up

		**DIME mean baseline (SD) (*n* = 8)**	**Control baseline (SD) (*n* = 6)**	**Adjusted mean difference (95% *CI*)**^**a**^	**Effect size (95% *CI*)**^**a**^
**PHQ9**	Baseline	8.00 (6.21)	11.33 (8.76)		
	6 months	5.71 (4.82)	8.67 (8.36)	− 1.60 (− 9.45 to 6.26)	0.02 (− 6.26 to 9.45)
	Mean change	2.29 (9.27)	2.67 (4.37)		
**PAID**	Baseline	32.50 (23.95)	46.67 (22.37)		
	6 months	27.14 (15.18)	30.42 (20.34)	2.95 (− 15.13 to 21.02)	0.013 (− 21.02 to 15.13)
	Mean change	6.61 (15.91)	16.25 (20.84)		
**ITAS negative**	Baseline	46.00 (10.84)	52.50 (10.84)		
	6 months	40.57 (6.90)	44.17 (8.98)	− 1.09 (− 10.29 to 8.10)	0.007 (− 8.10 to 10.29)
	Mean change	5.12 (10.45)	8.33 (8.98)		
**ITAS positive**	Baseline	14.63 (2.45)	14.83 (2.99)		
	6 months	15.00 (2.00)	13.17 (5.31)	1.70 (− 2.46 to 5.86)	0.076 (− 5.86 to 2.46)
	Mean change	0.00 (3.00)	1.66 (3.50)		
**ITAS total**	Baseline	55.38 (9.98)	61.67 (11.60)		
	6 months	49.57 (8.22)	55.00 (5.62)	− 2.45 (− 9.83 to 4.92)	0.052 (− 4.92 to 9.83)
	Mean change	5.14 (8.36)	6.67 (8.24)		

^a^Adjusted mean difference and effect size calculated using ANCOVA controlling for baseline

### Acceptability and process evaluation — exit interviews

All participants were invited for exit interviews, and 7 were conducted (control = 2 and DIME = 5) by RU and TS, who were independent of the intervention delivery team. All interviewees were optimistic about the group sessions indicating acceptability.

Thematic analysis of interview transcripts (by RU and TS) identified the following themes, social support and reflections on group size, the content of group sessions and experiences post-group. For the social support theme, both control and DIME group participants found the group mode of delivery helpful and learned and appreciated peer support:

Being in a group… [I] can appreciate everybody else’s position…you’re not dealing with it [insulin] in isolation (DIME participant, male, 50s).

Regarding group size, there were individuals from control and DIME groups who either preferred small groups or would have appreciated a larger group, indicating personal preferences. Views on sessions’ content differed as the content of control versus DIME sessions differed, with one control group participant indicating the need for a more ‘scientific’ explanation of why insulin was needed. In the DIME group, a participant reflected on the information she had received and how this helped improve her diet. Post-group experiences also differed with more challenges for control group participants, illustrated by a control group participant (female, 60 s) feeling “…confused…because I’m still trying to get my blood sugar lower…something’s not going right…”.

There were more positive reports for DIME participants, indicating changes to the morning routine:

[My routine] has changed a lot because now every morning I make sure it’s my duty to prick my finger and find out how much it is before I leave the house.”(Female, 60 s) Diet, “I go for the green leafy stuff, I don’t eat much meat, the grains …it’s really we talked a lot about.” (Female, 70 s) Exercise, “everyday I brisk walk for an hour.” (Female, 60 s) And there was less fear, “realising that well that’s just like taking any other drug, realistically, if [insulin] is what you need to take you take it. (male, 50 s).

### MI adherence

Intervention group sessions were audio-taped if study participants agreed to the sessions being audio-recorded. Control group sessions were not audio-recorded. An independent MI rater used the MITI to assess the interventionist’s skills in delivering the intervention using an MI mode of delivery and was assessed as achieving competency.

### Fidelity

Intervention fidelity was maintained with assistance from a research assistant (QT) who observed all DIME group sessions, assisted the interventionist and ensured that the curriculum was followed according to the suggested timings. This ensured that all the planned content was delivered. The exception was when the last group of participants had 2 sessions delivered solely by the interventionist by telephone during the UK’s COVID-19 lockdown.

## Discussion

This study aimed to determine the feasibility and acceptance of the group diabetes insulin self-management education (DIME) programme for people with T2D who are starting insulin. This pilot RCT describes the first UK programme for this population and is underpinned by behaviour change theory delivered using the principles of motivational interviewing. The main findings indicate that although recruitment to the trial was stopped early because of COVID-19, there was enough evidence to suggest that DIME is acceptable to people with T2D starting insulin and pre-COVID-19; it was feasible to deliver an intervention such as this. It was also feasible to recruit an ethnically diverse study population. These results suggest that the DIME could be used to develop a larger RCT to optimise insulin initiation for people with T2D.

Only 17 of the 60 planned recruits were randomised, and 14 completed the study. The sample size was not achieved because of COVID-19, and the UK’s Department of Health & Social Care stopped all research studies (excluding COVID-19 related) in March 2020. However, until then, recruitment was on track, and participation was higher than anticipated at 61%. Other pilot trials of people with T2D have had a lower participation rate [34]. Some people (*n* = 3) who were initially randomised to the study withdrew before the start of the programme, so no baseline data or questionnaire data was available. One participant did not return study questionnaires in the DIME group. Still, data was available for analysis for 13/17, indicating a retention rate of 76%, and 14/17 (82%) participants attended all study sessions.

Because of limitations owing to COVID-19, conducting a physical assessment for participants at 6-month follow-up was not possible, so only questionnaire data was available. The pilot study was not powered to detect any differences in outcome measurements, and the reliability of statistical tests used, i.e. ANCOVA, is limited due to the small sample size. However, there was a trend for all participants to have fewer negative beliefs of insulin (measured using the ITAS) once they had started insulin. This is a finding common to insulin treatment trials [21, 30]. Therefore, other measures of efficacy may be more relevant in this context, for example changes in confidence in self-managing insulin which may need to be developed for future study. A fully powered trial is needed to evaluate group differences.

The acceptability of the DIME intervention was determined via a semi-structured interview. All DIME participants who agreed to be interviewed indicated they found the DIME programme acceptable. This process evaluation also identified that DIME participants reported many positive ‘post group experiences’ following thematic analysis of the interview transcripts, including the session content on dietary strategies. The content was identified by people with T2D starting insulin in an earlier qualitative study to develop DIME [35].

The main limitations of this pilot trial are that we did not achieve the pre-specified sample size because of COVID-19. However, despite this, the recruitment rate before the UK lockdown was sufficient that the sample size would have been achieved with an additional 3–4 months of recruitment and the opening of the second recruitment site in Lewisham. The second limitation is that we were stopped from opening the second site, so all activities for the pilot occurred in Lambeth only. Having 2 sites would have given more information regarding the study process and data on the feasibility and acceptability of DIME. The third limitation was also a problem incurred because of COVID-19 that we could not conduct a face-to-face physical assessment and collect data on HbA1c and weight.

Future iterations of DIME and a full-scale trial to test the effectiveness of the intervention will need to understand more about the impact of COVID-19 in terms of the acceptability of face-to-face group education and explore alternative modes of delivery of the intervention. This may potentially involve a blended approach to intervention delivery. Future research in terms of refinement and further testing of DIME will need to consider the views of people with T2D who need insulin, the friends/relatives and carers who support them and the diabetes health professionals currently delivering insulin education.

## Conclusions

This pilot randomised controlled trial of the DIME intervention for people with T2D starting insulin has demonstrated that the intervention is feasible to deliver and acceptable to participants in South London, UK. The next step is to pursue a fully powered multisite randomised controlled trial to test the efficacy and cost-effectiveness of the intervention.

## Data Availability

The datasets used are available from the corresponding author on reasonable request.

## Abbreviations

T2D: Type 2 diabetes
DIME: Diabetes insulin self-management education
COVID-19: Coronavirus disease
OAD: Oral antidiabetic agents
UK: United Kingdom
NHS: National Health Service
HbA1c: Glycated haemoglobin
GP: General practitioner
LDICT: Lambeth Diabetes Intermediate Care Team
COM-B: Capability, opportunity, motivation-behaviour model
BMI: Body mass index
ITAS: Insulin Treatment Appraisal Scale
PAID: Problem areas in diabetes
PHQ-9: Patient Health Questionnaire
DSM: *Diagnostic Statistical Manual*
MITI: Motivational interviewing treatment integrity
CTU: Clinical trials unit
CONSORT: Consolidated standards of reporting trials
GLP1-RA: Glucagon-like peptide-1 receptor agonist
SD: Standard deviation
